# Study Designs to Assess Real-World Interventions to Prevent COVID-19

**DOI:** 10.3389/fpubh.2021.657976

**Published:** 2021-07-27

**Authors:** Jean C. Digitale, Kristefer Stojanovski, Charles E. McCulloch, Margaret A. Handley

**Affiliations:** ^1^Department of Epidemiology and Biostatistics, University of California, San Francisco, San Francisco, CA, United States; ^2^Department of Health Behavior & Health Education, School of Public Health, University of Michigan, Ann Arbor, MI, United States; ^3^Center for Vulnerable Populations at Zuckerberg San Francisco General Hospital and Trauma Center, University of California, San Francisco, San Francisco, CA, United States; ^4^PRISE Center (Partnerships for Research in Implementation Science for Equity), University of California, San Francisco, San Francisco, CA, United States

**Keywords:** COVID-19, study design, interrupted time series, difference-in-differences, sequential multiple assignment randomized trial, stepped wedge, preference design, implementation science

## Abstract

**Background:** In the face of the novel virus SARS-CoV-2, scientists and the public are eager for evidence about what measures are effective at slowing its spread and preventing morbidity and mortality. Other than mathematical modeling, studies thus far evaluating public health and behavioral interventions at scale have largely been observational and ecologic, focusing on aggregate summaries. Conclusions from these studies are susceptible to bias from threats to validity such as unmeasured confounding, concurrent policy changes, and trends over time. We offer recommendations on how to strengthen frequently applied study designs which have been used to understand the impact of interventions to reduce the spread of COVID-19, and suggest implementation-focused, pragmatic designs that, moving forward, could be used to build a robust evidence base for public health practice.

**Methods:** We conducted a literature search of studies that evaluated the effectiveness of non-pharmaceutical interventions and policies to reduce spread, morbidity, and mortality of COVID-19. Our targeted review of the literature aimed to explore strengths and weaknesses of implemented studies, provide recommendations for improvement, and explore alternative real-world study design methods to enhance evidence-based decision-making.

**Results:**Study designs such as pre/post, interrupted time series, and difference-in-differences have been used to evaluate policy effects at the state or country level of a range of interventions, such as shelter-in-place, face mask mandates, and school closures. Key challenges with these designs include the difficulty of disentangling the effects of contemporaneous changes in policy and correctly modeling infectious disease dynamics. Pragmatic study designs such as the SMART (Sequential, Multiple-Assignment Randomized Trial), stepped wedge, and preference designs could be used to evaluate community re-openings such as schools, and other policy changes.

**Conclusions:** As the epidemic progresses, we need to move from *post-hoc* analyses of available data (appropriate for the beginning of the pandemic) to proactive evaluation to ensure the most rigorous approaches possible to evaluate the impact of COVID-19 prevention interventions. Pragmatic study designs, while requiring initial planning and community buy-in, could offer more robust evidence on what is effective and for whom to combat the global pandemic we face and future policy decisions.

## Background

Most studies evaluating non-drug, large-scale behavioral interventions to prevent COVID-19 (e.g., shelter in place, social distancing, mask wearing, and school closure mandates) have been observational and ecologic, using group-level summaries rather than individual data. Early on in the pandemic, policymakers and scientists were forced to make rapid decisions in response to the evolving spread of SARS-CoV-2. Other than mathematical modeling, scientists and decision-makers primarily used available, aggregate data on disease incidence at the national and state and regional level. These data were analyzed to understand the effects of policy choices using designs such as pre-post (1) and interrupted time series (2–5), and analyses such as difference-in-differences (6–8). It was neither realistic nor ethical to attempt randomized controlled trials on a large scale to generate unbiased evidence about such policies. However, there are limits on the inferences that can be made from observational designs. All observational designs may be susceptible to unmeasured confounding, and it may be challenging to identify an appropriate control group. This is problematic because both factors can result in bias and it may not be possible to quantify its magnitude. Policy responses to the global pandemic often occur contemporaneously and separating the effects of various policies may be difficult or impossible. Further, most studies were ecologic and assessed, for example the effect of state-level policies on state-level incidence of COVID-19. This is not equivalent to the effect that a policy may have on an individual who adheres to it (termed the ecological fallacy). As one example, one could falsely conclude that face mask mandates do not lower COVID-19 transmission risk if one found an association between face mask mandates and higher incidence of COVID-19 at the state-level. In this case, the reason for such mandates may be the high incidence of disease when, in fact, wearing a face mask lowers an individual's risk of COVID-19 (9). In summary, observational designs are vulnerable to bias from multiple sources, some of which may be difficult to overcome given circumstances and available data.

Due to the limitations of these designs, it is imperative to begin designing and utilizing alternative study designs. Pragmatic studies could offer more robust evidence to inform decision-making as we face a mounting number of decisions about how and when to re-open aspects of public life (10, 11). They arose as a way to inform decision-making outside of the constraints posed by highly prescriptive clinical trials (12). Pragmatic studies are randomized or quasi-experimental studies whose goal is to provide evidence for implementation of an intervention into real-world practice. While reactive science (analyzing available data *post-hoc*) was appropriate for the beginning of the pandemic, it is important we now move to proactive science (planning and implementing *a priori* evaluations of interventions). Pragmatic study designs such as the SMART, preference, and stepped wedge designs could be used to evaluate school and restaurant re-openings and other community-level or clinic-based policy choices. Implementation-focused designs such as these offer greater design flexibility than traditional randomized trials and may achieve an important balance between internal (confidence in the causal relationships modeled) and external (ability to apply the conclusions outside the study) validity (13). Such designs require more initial planning than *post-hoc* analyses of available data and necessitate community engagement, but could in turn generate more robust evidence on what is effective and for whom to combat the global pandemic. For example, there have been uneven school re-opening plans, rollouts, and re-closings globally, indicating that some plans and efforts might be more effective than others (14–16). Pragmatic study designs could generate evidence on which are preferable and support equitable policy decision-making to aid communities in preventing COVID-19 and managing everyday activities such as schooling and work.

We aimed to interrogate published observational studies that examined policies to slow the spread of COVID-19. We identify strengths and limitations of each study design, and provide recommendations to improve validity of such studies. We then propose a suite of implementation-focused, pragmatic designs that could be useful to generate prospective data to support policy and decision-making in the future, noting examples of research questions they could be used to answer.

## Methods

We conducted a targeted (rather than a systematic) review of the literature to identify pertinent examples that were illustrative of different methodologies used to evaluate interventions and policies to prevent COVID-19. Our goal was to find instructive examples of common study designs for readers, rather than to comprehensively summarize the literature (which was rapidly changing). We focused our literature search on studies that evaluated the effectiveness of non-pharmaceutical interventions and policies to reduce spread, morbidity, and mortality of SARS-CoV-2. We searched on PubMed, Google Scholar, the United States National Library of Medicine LitCovid website (17), and in the references of identified studies. Search terms included SARS-CoV-2, COVID-19, shelter-in-place, stay-at-home, reopening, social distancing, mask, face covering, school, and education. For each article identified in our search, we characterized the study design. We hypothesized that the most common study designs to assess COVID-19 prevention would be pre-post, interrupted time series, and difference-in-differences and planned *a priori* to include these in the manuscript. We screened for other designs and pragmatic trials (e.g., stepped wedge designs), but found few or no examples of these at the time of our literature review. Given this focus on observational and pragmatic study designs, we excluded laboratory studies, surveillance studies, those focused on contact tracing, and modeling studies. We updated our search as we drafted the paper and met multiple times to discuss which studies to include. We chose to highlight studies for which methods were well-detailed and we could characterize aspects as particularly strong or weak. For each study design, we identified articles that we considered high-quality in that they included one or more aspect that strengthened their study design to address potential bias. Conversely, we also identified studies that demonstrated an obvious error or did not incorporate some of the available tools to strengthen inference. Juxtaposing aspects of study methodology was useful to inform ways to identify and address bias.

## Results

In the first section of this paper, we reflect on the existing literature regarding COVID-19 prevention (“Phase 1”) and use it to guide a discussion of the strengths and weaknesses of three study designs. In the second section, we propose study designs that could be used to study best practices for COVID-19 prevention in the future (“Phase 2”).

## COVID-19 Prevention Phase I: Observational Designs

Observational studies are often used to evaluate population health interventions for which experimental manipulation is not possible. Data is sometimes collected specifically for observational studies, but in the context of studies on the prevention of COVID-19, data on outcomes (and if possible, relevant covariates) were virtually all obtained through existing, external sources (e.g., routinely collected state COVID-19 incidence data). By analyzing data from before and after interventions, scientists try to isolate the effect of the intervention itself. Identifying a causal effect in practice, however, is difficult. One must wrestle with unmeasured confounding, trends over time (those naturally occurring or due to concurrent policy changes), and ideally, finding a control group similar to the intervention group on factors that influence the outcome of interest (19). Designs such as pre-post (a before vs. after the intervention comparison, usually with no control group), interrupted time series (a before/after comparison with extended time before and after, usually with no control group), and differences-in-differences (a before/after comparison with a control group) contend with these challenges slightly differently, and suffer from varying degrees of threats to validity. Here, we outline these observational designs and provide examples of COVID-19 related research studies that employed them. We also present strengths, challenges, and ways to improve the study designs used.

### Pre/Post

Pre-post studies compare the outcome of interest before and after the intervention (Table 1). They require a minimum of two timepoints and may or may not include a control group. The underlying assumption is, if not for the intervention, the outcome would have remained at the pre-intervention level. Thus, conclusions are susceptible to bias if anything else changes during the same time covered by the study period that affects the outcome.

**Table 1:** Overview of quasi-experimental designs.

**Design**	**Key design elements**	**Advantages**	**Disadvantages/threats to validity**	**Ways to strengthen**
Pre-post	•Comparison of outcome of interest before and after intervention. •May or may not include a control group.	•Less cumbersome and simpler to gather data for than other designs (requires data from a minimum of only 2 time points).	•Temporal biases are a key threat to validity; if there are changes in measurement or quality of data over time, this will cause bias. •Control groups, if included, may not be comparable for important covariates. •Concurrent policies challenge validity. •Lags in policy adoption can influence internal validity. •Infectious disease dynamics (e.g., exponential spread over time) can bias results.	•Include comparator groups. •Conduct adjusted statistical analyses. •Specify how time is being addressed in the design and analysis.
Interrupted time series (without control group)	•Data collected at multiple time points before and after an intervention is implemented. •Assess whether there is a level or slope change at the time of intervention (or after a pre-specified lag, if appropriate).	•Each group acts as its own control. •May be only option for studying impacts of large-scale health policies when there are no groups left unexposed to intervention.	•Requires a large number of measurements. •Preferred to have more pre-period data collection. •Relies on the assumption that nothing changed within the study period that would affect the outcome of interest other than the intervention. •Concurrent policies can influence results. •Temporal issues & seasonality are major challenges. •Lag periods must be appropriately conceptualized. •Infectious disease dynamics, such as non-linear functional forms, can bias results.	•Include comparator groups •Ensure adequate number of time points pre- and post-intervention (having sufficient data prior to the intervention will establish existing trends). •Conduct adjusted statistical analyses, with adjustments for time to reduce biases related to seasonal variability. •Adjust for autocorrelations. •Shorten the duration of time periods.
Interrupted time series (with control group)	•Data collected at multiple time points before and after an intervention is implemented in a treatment group and control group. •Most commonly analyzed using a difference-in-differences approach. •Compares the difference in the amount of change in the outcome before and after an intervention is implemented between groups exposed and unexposed to the intervention.	•Controls for observed and unobserved time-invariant variables that differ between groups.	•Requires a large number of measurements. •Preferred to have more pre-period data collection. •Relies on the assumption that nothing changed within the study period that would affect the outcome of interest other than the intervention. •Concurrent policies can influence results. •Temporal issues & seasonality are major challenges. •Lag periods must be appropriately conceptualized. •Inference relies on parallel trends assumption being met.	•Evaluate parallel trends assumption. •Use event-study design that estimates intervention effect at multiple time points before and after implementation (to check for bias and changes over time).

Perez-Lopez et al. (1) used an uncontrolled pre-post design to test for differences in the amount of respiratory illness in a pediatric emergency department in Qatar before and after school closures in response to the pandemic (Table 2). They compared the average proportion of positive tests for illnesses such as influenza A, adenovirus, and common human coronaviruses before the school closure (February 12-March 14, 2020) to the average after school closure (March 15-April 11, 2020). They correctly included a short lag period to take into account the incubation period of influenza A (although, it is unclear whether any of the other viruses have longer incubation periods). The authors were particularly interested in influenza A because antigenic drift and shifts result in a large pool of people without pre-existing immunity, just as the population was naïve to SARS-CoV-2. They concluded that there was a reduction in influenza A and adenovirus transmission. However, inference from simple pre-post studies carries a number of limitations. School closure was the first social distancing measure implemented by the government of Qatar. If any other policies were implemented in this period, the effects seen may not be attributable to school closure alone. Further, if usually there are seasonal trends during this time, decreases due to this could be falsely attributed to school closure. A strength of the paper is that the authors did a falsification test, comparing rates of influenza A during the study period to the same weeks in 2019 to demonstrate that seasonality is unlikely to be the explanation for the decrease. Bias from seasonal trends could also be mitigated by inclusion of a control group whose schools remained open in the post-intervention period.

**Table 2:** Selected examples of quasi-experimental studies evaluating real-world interventions to prevent COVID-19.

**Policy/intervention**	**Example**	**Strengths**	**Weaknesses**	**Ways to strengthen**
**Pre/Post**				
School closures (Mandate by the Qatari government)	•Compared rate of positive tests for respiratory viruses other than SARS-CoV-2 in a pediatric emergency department before and after school closures in Qatar (1).	•Specified lag period for influenza A. •Compared to trends in 2019 to rule out that seasonal variations could explain the results.	•No control group. •Only captures children who were sick enough to go the emergency room (problematic, for example, if proportion of children with influenza A whose parents take them to the hospital changed over time). •Depending on the hospital catchment area, may be unclear who is in the target population the study sample represents. •Unclear what other interventions or policies to reduce spread of COVID-19 were enacted during the study.	•Comparison group would improve validity. •Consider stratification on relevant characteristics (i.e. age group).
**Interrupted Time Series: Without Comparison Groups**				
Physical distancing (Closures of schools, workplaces, and public transport, restrictions on mass gatherings/public events, and restrictions on movements [lockdowns])	•Assessed incidence of COVID-19 before and after implementation of physical distancing interventions in 149 countries or regions, synthesized using meta-analysis (4).	•Compared effect of five physical distancing interventions overall and in smaller subsets of policies to attempt to determine the most effective combination and sequence. •Specified lag period a priori. •Restricted post-intervention period to address temporal concerns and reduce bias given limited pre-intervention time period. •Allowed for country-level variation using random effects models in random effects meta-analysis to synthesize effect estimates. •Assessed and controlled for country-level characteristics.	•No control group that was not subjected to at least one intervention.	•Comparison of “similar” clusters of countries (i.e., East African nations, Scandinavian nations) could improve analyses & interpretation.
Mask mandate (Universal mask wearing required by health system for healthcare workers and patients)	•Compared SARS-CoV-2 infection rate among healthcare workers before and after implementing universal masking in one health care system in the US (5).	•Allowed for non-linear functional form of SARS-CoV-2 positivity rate.	•Testing was implemented for healthcare workers, but didn't fully account for lags in development of symptoms after implementation of policy in their division of time. •Didn't account for statewide trends (e.g., the reduction observed could be due to other policies outside the healthcare system). •External validity is a concern—healthcare workers not generalizable to other high-risk exposure settings (e.g., food service sector jobs).	•Add comparison group. •Would benefit from statistical adjustment for other interventions external and internal to the hospital. •Analyzing trends during the implementation period could assist with assessing changes in slopes/trends.
Social distancing measures (closures of schools, closures of workplaces, cancellations of public events, restrictions on internal movement, and closures of state borders)	•Estimated change in COVID-19 case growth and mortality before and after implementation of first statewide social distancing measures (2).	•Specified an event-study design as a robustness check. •Conducted sensitivity analyses with multiple incubation periods and to address weekly periodicity.	•The type of the first social distancing measure may have differed across states. •It is not possible to identify which policy was most effective. •Biased if amount of testing (and therefore identification of cases) differed before and after intervention.	•Exploration of how lifting of policies, as compared to those who kept policies (i.e., duration of intervention), could improve interpretation. •Imbalance of time between the pre- (17 days) and post-periods (25 days); post-period is longer than pre-period.
School closures (State government mandates)	•Assessed whether school closures impacted incidence of COVID-19 at the beginning of the pandemic in the US (3).	•Included other non-school related policies (e.g., stay at home orders) in models. •Clear justification for lag period and conducted sensitivity analyses with multiple lag periods. •Adjusted in models for important covariates, such as testing rates, urban density, comorbidities, and age. •Included interaction effects between school closure & covariates.	•No control group. •Median time from school closure to last enacted other intervention was 5 days in states in highest quartile of COVID-19 incidence at time of school closure and 12 days in lowest quartile of incidence—may be difficult to separate out effects of other interventions, despite controlling for them.	•Localized nature of policies could provide advantage for cluster ITS comparisons, as compared to state-level data used in the study. •States implemented other interventions at the same time as or shortly after school closures, making it difficult to completely isolate the effect of school closure, despite controlling for other interventions.
**Interrupted Time Series: Integrating Comparison Groups**				
Stay-at-home orders (State government mandates)	•Compared COVID-19 cases in border counties in Illinois (where a stay-at-home order was issued) to border counties in Iowa (where such an order was not issued) (6).	•Comparison of border counties potentially less likely to be biased than comparison of larger geographic area. •Sensitivity analyses to account for differences in timing of closing schools/non-essential businesses and to assess whether there were differential trends by population density and poverty rates.	•Only one pre-period, as compared to six post-periods.	•Inclusion of analyses of sequencing of orders in Iowa could strengthen analysis. •Control for county-level COVID-19 testing trends.
Social distancing measures (Bans on large social gatherings; school closures; closures of entertainment venues, gyms, bars, and restaurant dining areas; and shelter-in-place orders)	•Assessed effect of social distancing measures on measures of growth rate of confirmed COVID-19 cases in US counties using an event study design (7).	•Event study design (including fixed effects for county and time) allowed testing of parallel trends assumption in pre-policy period. •Tried to separate out effects of different policies. •Multiple robustness checks.	•Relying on administrative boundaries such as counties may not reflect how people live their lives (e.g. working across county lines), making it more difficult to interpret findings. •Longer post-period, as compared to pre-period.	•Could have used localized data to make comparisons over time, comparing similar states (clusters) with more or less restrictive orders. This is particularly important given that controlling for number of tests was done at the state-level, not locally. •Extension of study period after April 27, when orders were being lifted could have provided additional evidence of changes. Particularly of concern given that April 7th was when 95% of the U.S. population was covered by shelter-in-place orders.
				•Inclusion of a longer pre-intervention period would improve the study; could have used excess mortality as a marker of COVID-19 cases. •Could have used state politics as a covariate, which influences policy decision making.
Face mask mandates (State government policies to wear face masks or covers in public)	•Assessed effect of state government mandates for face mask use on changes in daily US county-level COVID-19 growth rates using an event study design (8).	•Event study design allowed testing of parallel trends assumption in pre-policy period. •Compared state-wide face mask mandates and employee only mandates. •Controlled for other policies implemented (e.g., social distancing policies) and state-level COVID-19 tests, including growth rate. •Adjusted for other state characteristics (e.g., population density). •Multiple robustness checks.	•Some states did not have state-wide mandates, but counties within them enacted mandates. •Few data points available pre-intervention.	•Local-level variation in adherence to mandates could alter results, comparison of county adherence measures (e.g., fines) could strengthen analyses.

### Interrupted Time Series: Without Comparison Groups

**Figure 1:**
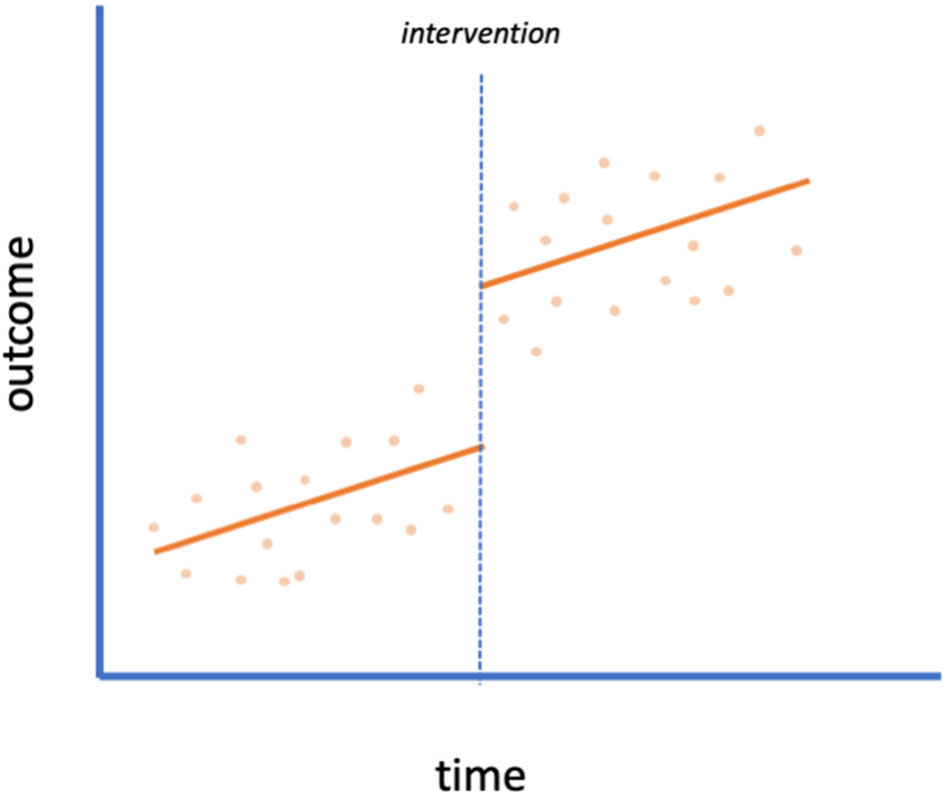
Interrupted time series. An example of an interrupted time series design with no control group. A scatterplot of data is shown with the intervention implemented at the time of the dotted line. This plot indicates a level change (but no slope change) due to the intervention.

Interrupted time series (ITS) designs aim to identify the effect of an intervention by using data at multiple time points before and after its introduction (Figure 1). ITS is one of the most commonly used approaches to evaluating policy interventions (18). This design compares the level and trends of the outcome present before the intervention to the level and trends after introduction within a group using a segmented regression model. The assumption is that the pre-trend would continue unchanged in the absence of the intervention. Change in the observed outcome in level or slope is attributed to the intervention (13, 20). Using data from multiple time points pre- and post-intervention makes ITS a stronger design than a simple pre-post comparison without a control group (with data from one time point pre- and one time point post-intervention). It is important with ITS studies to have enough time points of data (particularly in the pre-period to establish the pre-intervention trend) to make comparisons. Given the novelty of COVID-19, studies completed at the beginning of the pandemic utilizing ITS could be impacted by limited pre- and post-intervention data. Lastly, concerns about temporal trends, such as seasonality, may exist, especially in the face of transmission dynamics of infectious disease.

Once more, when interpreting results, it is important to consider that factors other than the intervention could be the cause of any observed changes in the outcome (13). In the case of COVID-19, many policies were enacted on a large scale within a compressed time period (e.g., school closures and shelter-in-place). It may be difficult to disentangle the effect of each individual policy (2, 3, 9). Auger et al. (3) assessed whether US school closures were associated with decreased COVID-19 incidence and mortality at the onset of the pandemic using ITS models. The study's authors included covariates such as other state-level policies, testing rates, and demographic characteristics to attempt to isolate the independent effect of school closures. Yet, the authors noted that the median time from states' school closure to their last enacted non-pharmaceutical intervention ranged from 5 to 12 days. States with lower COVID-19 incidence at the time of school closure were found to have the largest relative reduction in incidence due to school closure. However, these same states also had longer implementation periods of other non-pharmaceutical interventions. Given the concurrent nature of many interventions, it is difficult to conclude that the estimated effects solely represent school closure.

Similarly, Wang et al. (5) concluded that universal masking in a healthcare system was associated with a significantly lower rate of SARS-CoV-2 positivity among healthcare workers. However, the effect of mask wearing (implemented March 25 for healthcare workers and April 6 for patients) is impossible to fully disentangle from the many other precautions put in place by the health system during the study period (e.g., restricting visitors, stopping elective procedures, limiting on-site work) as well as state policies (the Massachusetts stay-at-home order was implemented March 24). Inclusion of a control group could help strengthen this design. For example, if another hospital in Massachusetts did not implement a universal masking policy and had higher SARS-CoV-2 positivity among its workers, that would strengthen the conclusions of the study. Yet, even if a control group is included, there is still a risk of bias if factors that influence the outcome, other than the intervention, are not comparable between the groups. To alleviate lack of comparability, it might be feasible to find a control hospital that matches characteristics of the implementing hospital that may influence the outcome of interest.

In another study, Islam et al. (4) explored packages of interventions (rather than individual policies), reflecting the reality of many communities' efforts to prevent the spread of COVID-19. They used data from 149 countries with different combinations of policies (closures of schools, workplaces, and public transport, restrictions on mass gatherings and public events, and restrictions on movement [lockdowns]) to attempt to determine which combinations and sequences of policies worked to decrease incidence of COVID-19 using ITS. Overall, they found that physical distancing interventions were associated with reductions in COVID-19 incidence globally. Earlier implementation of lockdown was associated with a larger reduction in COVID-19 incidence. By assessing the effect of bundles of interventions, the authors solved the problem of mistakenly concluding an intervention had an effect when it was actually caused by a concurrent intervention. However, this approach renders it difficult to identify the effect of each specific component within the bundle. (Islam et al. was able to draw conclusions about the effect of public transit, for example, because it was included in some countries' responses and not in others). Further, ITS depends on a sharp disruption in the outcome to correctly estimate the effect. When the intervention is phased in over time (as is likely with multiple components), the effect is more problematic to identify and more susceptible to errors in model specification.

With behavioral policies in particular, the date of initiation by the government may not reflect the public's behavior in practice. It could take days to weeks for the majority of behavior change to manifest or, alternatively, people could take certain precautions before a policy is officially enacted. This issue is compounded by the fact that with an infectious disease such as COVID-19, there is a lag from when behavior change (e.g., mask-wearing) may affect transmission to when a change in outcome would be observed (e.g., symptomatic cases or deaths). It is important to specify the expected lag a priori (3, 4); letting the data determine the impact model [as in Voko and Pitter (21)] could lead to spurious conclusions (9, 20). Sensitivity analyses varying the lag period can also demonstrate robustness of results [e.g., Siedner et al. (2), Auger et al. (3), Islam et al. (4)]. Furthermore, it is useful to provide graphs displaying fitted models and underlying data (as in Figure 1) so that model fit can be assessed (2).

### Interrupted Time Series: Integrating Comparison Groups

**Figure 2:**
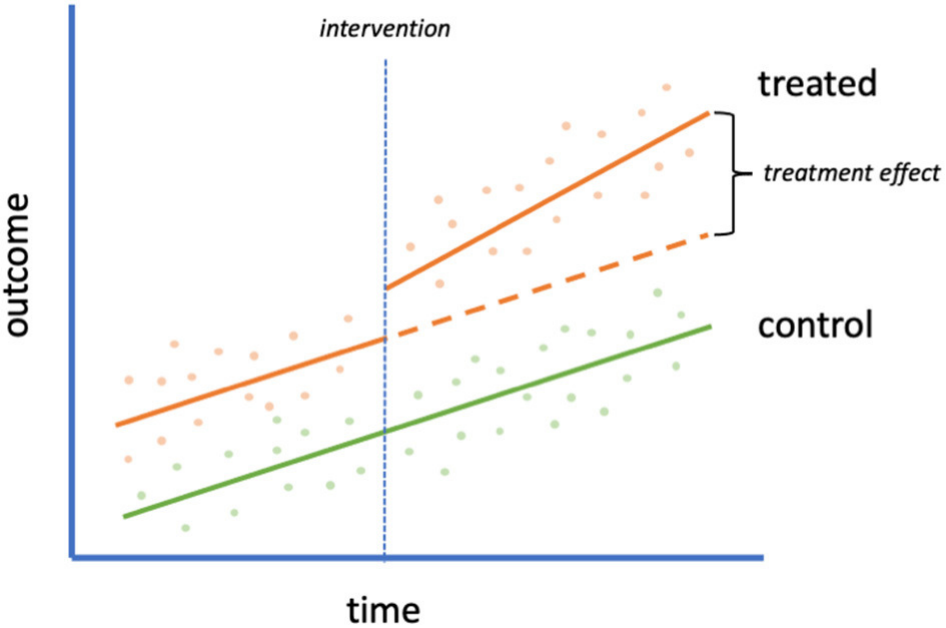
Interrupted time series with control group. An example of an interrupted time series design with control group (often analyzed with a difference-in-differences approach). A scatterplot of data is shown with an intervention (orange) and control (green) group. The intervention is implemented in the treated group at the time of the vertical dotted line. The orange dashed line refers to the hypothetical outcome of the treated group in the absence of the intervention. The difference between this hypothetical outcome and the actual outcome is the treatment effect.

Time-series data with a control group are commonly analyzed with a difference-in-differences (DID) approach. In this design, the effect of an intervention is explored by examining the level and trend of the slope of an outcome before and after the intervention, comparing them across treatment and control groups (Figure 2). In a time-series design with a control group, it is assumed that the treated group would have the same trends over time as the untreated group were it not for the intervention (the parallel trends assumption). The DID method controls for variation in fixed characteristics, both observed and unobserved, making it less prone to unmeasured confounding than some other methods (18).

To maximize comparability between treated and untreated groups, Lyu and Webhy (6) assessed the effect of a stay-at-home order on estimated rates of COVID-19 within contiguous counties. By isolating to a small geographic area, the treated (border counties in Illinois) and untreated groups (border counties in Iowa) were theoretically more similar than if they were sampled from a larger and more heterogenous area. The authors added county-specific fixed effects to control for county time-invariant differences. Results demonstrated that Iowa had higher rates of COVID-19 cases than Illinois following Illinois's stay-at-home order. To evaluate the parallel trends assumption, Lyu and Webhy assessed whether trends in the outcome before the stay-at-home policy went into effect in Illinois were similar to those in Iowa and found they appeared to be. Additional robustness checks can boost confidence in the results of a DID model. When comparing border counties, Lyu and Wehby (6) did sensitivity analyses to account for differences in timing of closing schools and non-essential businesses and to assess whether there were differential trends by population density and poverty rates. Similar methods could be used to evaluate restrictions globally with varied policies across geographies and at different time points, e.g., in the United Kingdom where different regions were placed into tiers based on incidence of infection and subjected to varying levels of public health restrictions (22) or as the previously described study by Islam et al. (4) that explored packages of interventions in 149 countries.

Another analytic option for time-series data with a control group is an event study model (23). These are a more flexible version of a traditional DID that interacts policy variables with multiple indicators of time before and after implementation. Specifying such models may allow assessment of assumptions (e.g., reverse causality and whether pre-intervention trends are parallel) and determination of how policy effects change over time (23, 24). Courtemanche et al. (7) note that an event study design's ability to reveal trends in intervention effects over time is particularly useful to study lagged outcomes such as COVID-19 incidence. They used an event study regression to examine the effect of social distancing measures to slow the spread of COVID-19. They estimated the separate and combined effects of four county-level social distancing policies by estimating one model that included variables for each policy and its variation over time. Their models displayed an increasing effect of government-imposed social distancing measures over time.

Event study designs allow for control of known and unknown time-invariant differences, as well as known time-varying differences between treatment and control groups. Interventions that may affect the outcome (other than the treatment of interest) must be controlled for in analyses if they are introduced differentially in either the treated or control groups during the study period (23, 25). Lyu and Wehby (8) used such an event study design to assess the effect of state government mandates to wear face masks in public on the daily county-level COVID-19 growth rate, examining how effects changed over five post-intervention periods. They controlled for time-varying differences in other mitigation and social distancing policies between states and counties to isolate the effect of face mask mandates. They concluded that requiring face mask use in public could help to mitigate spread of COVID-19.

To bolster their conclusions, Lyu and Wehby (8) and Courtemanche et al. (7) also executed multiple robustness checks. In both studies, authors estimated various alternative specifications of their model and confirmed the general pattern of results was similar. While such sensitivity analyses are useful, if robustness checks suffer from the same biases as the primary analysis (e.g., uncontrolled time-varying confounding), they may only serve to reinforce biased findings.

## COVID-19 Prevention Phase II: Implementation-Focused Pragmatic Designs

In this section, we outline a series of pragmatic study designs that could be used to gather data prospectively. Pragmatic trials aim to assess the effectiveness of interventions or policies in real-world practice (as compared to classic, explanatory randomized controlled trials that aim to assess efficacy under idealized conditions) (12, 26). The goal for a pragmatic study is to maintain the quality and strength of the comparisons that randomizing treatment provides (although, some may be quasi-experimental), while implementing the intervention in a realistic setting to populations that are representative of those who would receive such an intervention if it was provided in usual care. By giving priority to implementation processes, these implementation-focused designs can enable a deeper understanding of factors that increase or interfere with implementation success. These designs explicitly focus on external validity to be able to generalize the findings in ways that support translation and application of the findings into practice (the focus of implementation science), as compared with traditional randomized controlled trials that focus on internal validity (27). With lessons about uptake from implementation-focused study designs, e.g., across different types of intervention sites, or by population groups, it is possible to clarify where additional intervention efforts may be needed, to increase overall uptake and to ensure equity across intervention areas. We offer examples in Table 3 of how each design could be applied to evaluate interventions for COVID-19 in the community.

**Table 3:** Pragmatic study design examples applied to community interventions during COVID-19.

**Study design**	**Implementation problem the design can address**	**Example**	**Outcome and comparisons**
Two-stage randomized preference	Health departments would like to offer incentives to contacts to remain in quarantine for the full recommended duration but cannot offer all types of possible incentives so would like to determine which are more impactful.	**CONTACT TRACING PROGRAM INCENTIVES TO QUARANTINE**Choices about different incentives to stay in quarantine for the full recommended duration may impact uptake of strict adherence to health department recommendations for contacts identified in contact tracing programs. Understanding to what extent there are benefits of different preferences on outcomes for different types of incentives can help programs plan for the highest impact. A two-stage randomized preference trial can help answer these questions. **Example Research Questions:** •*Does randomizing contacts to receive a cash stipend, a package of resources or a choice between the two result in greater proportion of contacts staying in quarantine?* •*Does randomizing contacts to receive a cash stipend, a package of resources or a choice between the two result in different COVID-positive test probabilities after the quarantine is over?* •*How different is the uptake of these two approaches among contacts randomized to choice? What about sub-groups of interest (e.g., by age group, ethnicity, or employment status)?*	•Participation/engagement levels for those randomized to different options vs. randomization to preference. •Impact of randomization vs. choice on self-reported or test positivity outcomes.
SMART	Clinic systems may not be able to offer video-visits to all patients, and can benefit from determining whether less intensive formats (e.g., telephone calls; email communications) are sufficient for some patients, allowing the more intensive formats to be offered to those who struggle with other formats, or whose health needs do not align with less intensive formats.	**CLINIC-BASED TELEMEDICINE**Clinics (or individual providers) are randomized to one of two telemedicine approaches for registered patients (e.g., telephone visit or video visit). Those who are not engaging with options after a specified period of time, are re-randomized at an intermediate point to either the other intervention or an augmented form of care, such as a health coach call. **Example Research Questions:** •*Are patients (or providers) given (a) a single intervention A or B equally likely to complete follow-up recommendations (e.g., labs, medications refills) as those given (b) a sequenced combination of the two (A and then B, or B and then A) or (c) an augmented intervention, such as A plus an augmented form of care?*	Comparisons of: (1) patient-level and provider-level engagement with different telemedicine options; (2) levels of satisfaction; (3) outcome metrics such as completion of referrals, labs, refills of patients in different groups/no-show rates at the clinic.
Stepped wedge design (modified)	Schools may want to re-open but prefer a staggered approach, in which all schools start with on-line learning, and then depending on outcomes of COVID-19 testing after the school starts, changes in restrictions are made, such as in-person attendance.	**STAGGERED IMPLEMENTATION OF IN-PERSON SCHOOL WITH TEST-BASED DECISION-MAKING AT EACH STAGE**By using a combination of a stepped wedge design (with staggered roll-out) plus modifications to the intervention at pre-specified time points as in a SMART design, a staggered modifiable implementation of school sites for in-person classes can be evaluated. For this design, at the end of each set time period, the COVID-19 prevalence is estimated and decisions about how to either stay in the most restrictive mode or to advance to a less restrictive approach are evaluated. A staged approach to testing different educational environments a school district may allow for alterations to restrictions as each new phase is rolled out.	•Do the schools/classrooms meet the advancement criteria for moving to the next school reopening level? •Adjustments to the school environment, such as outdoor classrooms, time spent in class, ventilation, classroom student numbers, etc., can also be incorporated to see if there are additional impacts on prevalence, if these options are feasible.

### Preference Designs

When the primary objectives of the study are to disseminate interventions widely (rather than focusing specifically on efficacy), preference (or “choice”) designs can be considered. There are two types of preference designs that allow for some level of patient-directed preference, partially and fully randomized preference trials. Partially randomized designs (not covered here) are mostly intended to improve participation among sub-groups who might otherwise refuse participation. While this can be an important study objective, it can complicate interpretation of study findings across the preference and randomized groups. With fully randomized designs, participants are randomized to one of two or more interventions and a choice arm. This allows for estimation of the impact of having a choice of treatment modality on study outcomes, particularly those that may be considered “preference-sensitive.” In the context of COVID-19, e.g., some Black parents have decided to keep their children at home, rather than choose in-school learning opportunities, because of the inequitable burden of COVID-19 infections (28). Studying different schooling models with a preference design could enable one to measure the effect of parents' preferences on outcomes such as children's well-being and learning.

**Figure 3:**
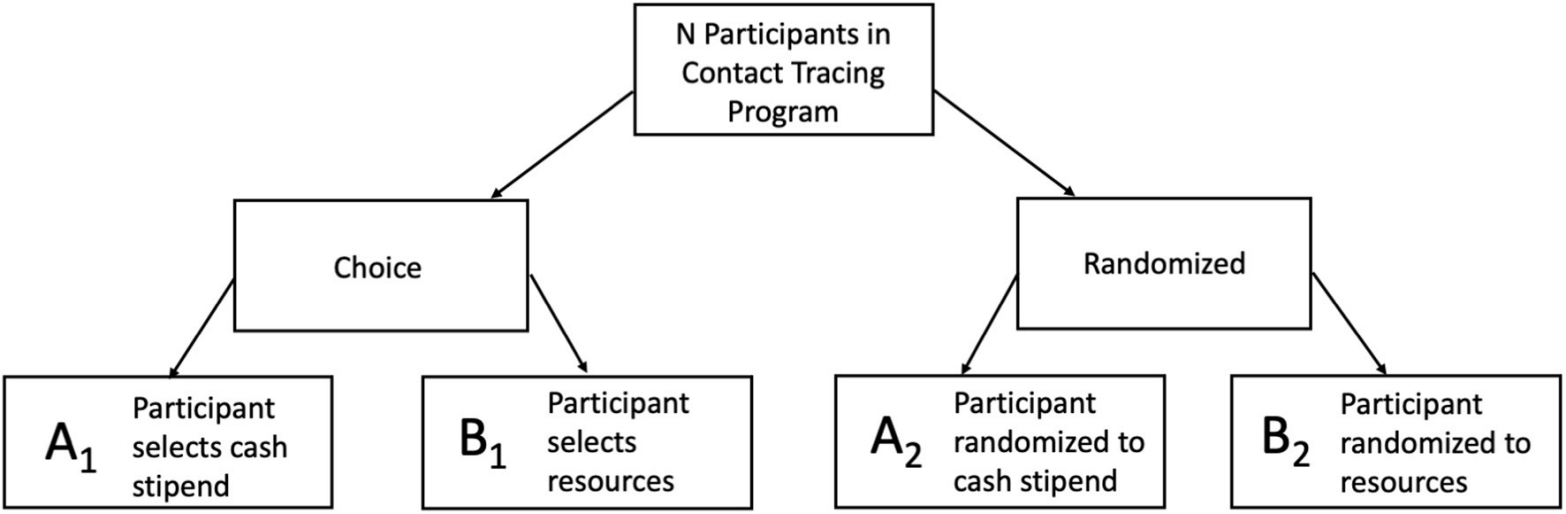
Two-Stage Preference Design for Contact Tracing Quarantine Incentives. The comparison of uptake of A_1_ vs. B_1_ shows the selection effect. *Is there differential uptake of these two programs?* If yes, then there is a difference in the groups' overall selection likelihood. The comparison of outcomes of A_2_ vs. B_2_ shows the difference between two programs through a controlled trial design. For example, for the research question: *Is there a difference in measures of successful completion of quarantine between the two programs?* The comparison of outcomes of A_1_ vs. A_2_ and B_1_ vs. B_2_ shows the preference effect. For example, if more participants who selected cash stipend (A_1_), were likely to complete their second COVID-19 test than those who were randomized to cash stipend (A_2_).

In the two-stage randomized preference design full randomization occurs at the outset of the study, with individuals or sites randomized (usually with equal probability) to one of two or more intervention arms or to an arm that offers a choice between the interventions. This design allows for examination of important differences between choice arm outcomes and non-choice arm outcomes in situations where randomization is appropriate. When there is a group randomized to a choice arm, it is possible to examine the impact of preference-based selection compared to randomization to each intervention option, for outcomes like adoption/adherence or reach within a particular population of interest (see Figure 3). For example, a COVID-19 prevention intervention focused on application of different types of incentives to increase uptake of self-isolation/quarantine behaviors, could be studied with a preference design (Table 3). The goal of the study might be for a health department to determine whether offering cash incentives or vouchers for food and services increases adherence to recommendations, among contacts reached through a contact tracing program. A small-scale study of this kind, among targeted groups or a random sample, could provide useful information on which option has higher uptake, and the association of each type of incentive with adherence outcomes.

This design can also be applied to clusters and situations where stratification on important individual or group characteristics is desired. An assumption is that prospective participants are familiar enough with intervention content to be able to have a preference, even if they are randomized to not choosing, or that informed decision aids are included in the study. Because of the randomization equity between the intervention arms and the choice arm, a few interesting responses from participants can be measured (29). First, consider the difference in effect size for participants who chose intervention A or B vs. participants who were randomized to intervention A or B (e.g., did offering a preference result in differences in outcomes compared with those from the randomized non-preference groups?). This is called a *preference effect*, the additional change in outcome that results from the interaction between a participant's preferred intervention and the intervention he/she actually receives (e.g., among those who receive intervention A, how different are the outcomes in the group that chose intervention A compared with the group that was randomized to intervention A?). One can also examine the effect of a participant's selection on outcomes, considered the *selection effect* (e.g., among those in the choice arm, how different are outcomes in the group that chose intervention A compared with the group that chose intervention B?). This is evaluated by determining whether participants preferring one intervention have differential responses compared with participants preferring another intervention. A limitation of these designs is that it is possible that those randomized to the choice arm will have unbalanced preferences, and this can constrain interpretation of findings and the ability to compare across groups.

### Sequential Multiple Assignment Randomized Trial Designs

The sequential multiple assignment randomized trial (SMART) involves an initial participant or group (e.g., classroom or clinic) randomization and follow-up period after which the uptake of the intervention or intervention component is evaluated. The logic of the design is that there are often multiple components in interventions and the best way to sequence them may vary. As well, some components may have higher or lower yield than initially hypothesized and knowledge of their uptake can inform implementation and determine the best use of resources (30).

**Figure 4:**
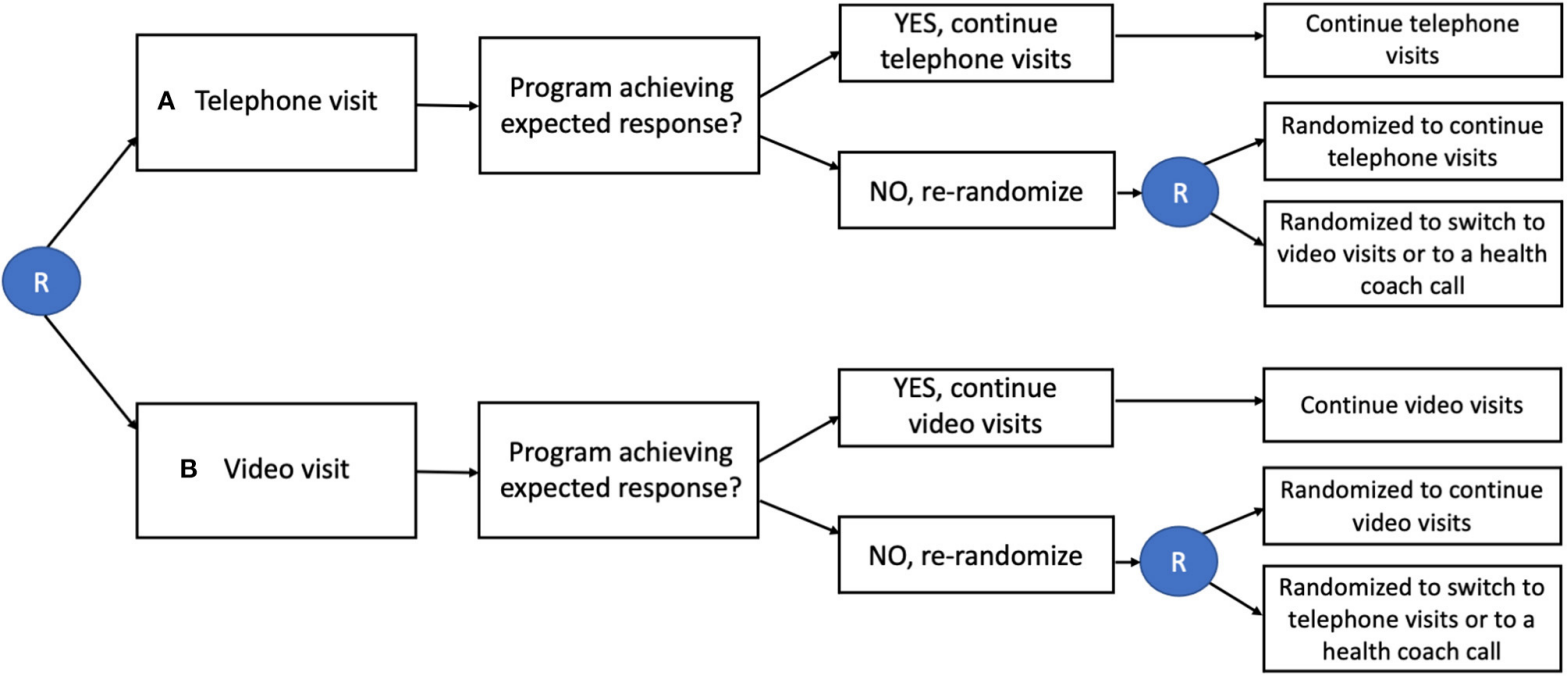
SMART Design for Telemedicine Visit Type in Primary Care. Individuals are initially randomized (R in circle) to either telephone visits or video visits. Those who are not responding to the intervention are re-randomized to continue the same intervention, switch interventions, or add a health coach call.

With this design, if uptake of an intervention component is low or inconsistent (such as inadequate social distancing, or mask wearing) or the outcome is poor (e.g., high transmission rate), the individual (or group) is then re-randomized to either a different intervention or continuation of the initial intervention (see Figure 4). Those who initially were randomized and are determined to be adherent to the intervention (or achieve a minimum criterion for a primary outcome) are not re-randomized or could be re-randomized to a less-intensive intervention. Studying the uptake of different components in an initial time period, and then adjusting the intervention to respond to the results, allows for a thorough evaluation of best components and sequencing for different components. Additionally, this second level of randomization utilizing responses from the first phase can allow: tailoring, intensification, augmentation, or replacing intervention strategies – improving efficiency and focus. The SMART trial approach is based on the prediction that individuals will differ in their response to an intervention and that as a result, will require either a step-up, step-down or switch in intervention components. A SMART design involves randomizing participants to intervention options generally according to pre-specified probabilities, often in an attempt to achieve balance in sample sizes across possible treatment sequences, even though this cannot be guaranteed (31). One additional advantage may be that SMART designs can encourage participation, in that changes may occur after an initial phase. For example, schools could start off with intensive social distancing protocols, and then investigators could re-randomize schools to de-intensification of measures (e.g., decreasing social distancing distance from 6 to 3ft) among those that have successfully kept cases and/or transmission down. SMART trial designs involve multiple points of assessment of intervention uptake, and as a result, can be at risk for information bias. For example, staff's knowledge of both initial treatment assignment and the value of the tailoring variable might influence the assignment process and lead to differential assessment of participants (32). Other challenges involve getting the timing right for when to make assessments of uptake and consider re-randomization of participants into tailored, enhanced, or other intervention adjustments.

### Stepped Wedge Design

**Figure 5:**
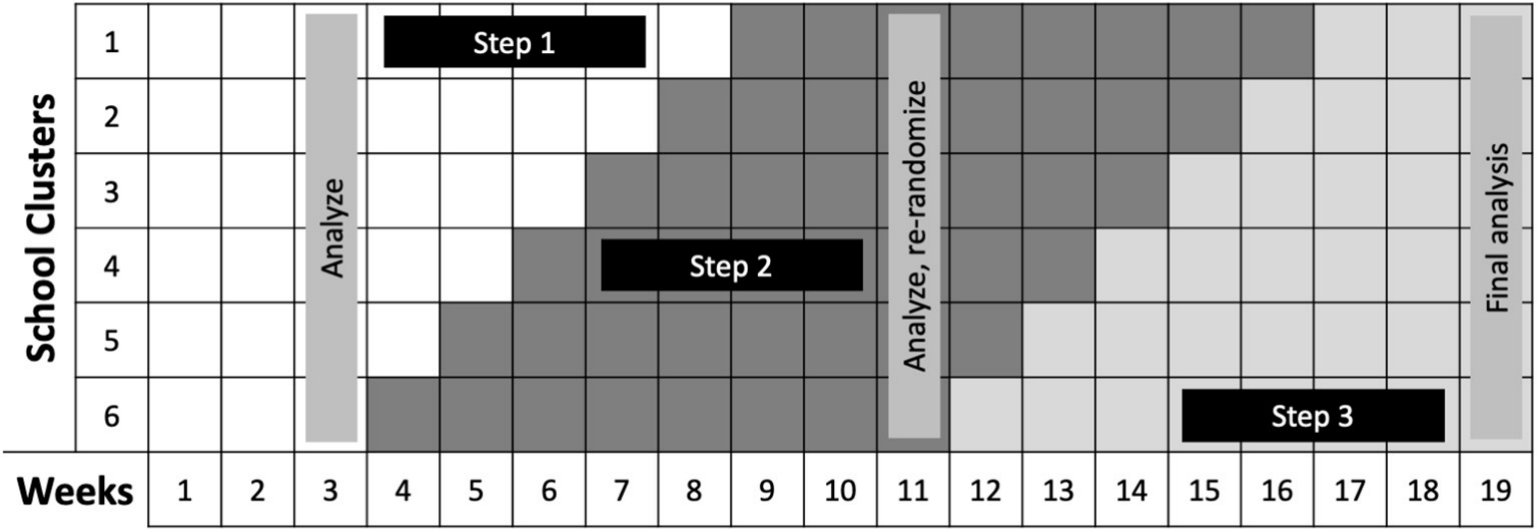
SMART/Stepped Wedge Design for School Re-Opening. Credit: Dr. Naomi Bardach, University of California, San Francisco. In this design, Steps 1–3 each represent an increasing number of in-person students. The team will conduct baseline: (1) PCR COVID-19 testing at all schools, for students and teachers and staff, and (2) student and teacher surveys regarding exposure and symptom history. Then, weekly PCR testing for a random sampling of students and staff within each school cluster will be conducted to determine if changes from Step 1 to Step 2 will be allowable after 3 weeks. If no new outbreaks occur during the move to Step 2, nor during the weeks 9–11 when all schools are in Step 2, all school clusters will be newly randomized and move to Step 3 practices. If no or limited outbreaks occur, we will recommend staying in Step 3 restrictions. Should there be large outbreaks or several small outbreaks in any of the schools in any of the stages, schools can return to the more restrictive Step 2 practices.

With the stepped wedge design, the intervention is rolled out over time, usually at the site- or cluster-level, allowing for staggered implementation. Participants/sites not receiving the intervention initially subsequently cross over to receive the intervention (33). In this design there is a one-directional rollout over time of an intervention. Initially, all clusters (or individuals) are unexposed to the intervention, and then, at regular intervals, selected clusters cross over (or step) into a time period where they receive the intervention (see Figure 5). All clusters receive the intervention by the last time interval (although, not all individuals within clusters necessarily receive the intervention). Data are collected on all clusters such that each cluster contributes data during both control and intervention time periods. The order in which clusters receive the intervention is ideally assigned randomly, but investigators may use another approach when randomization is not preferable or feasible. For example, in settings with geographically remote or difficult-to-access populations, a non-random order can maximize efficiency with respect to logistical considerations. However, this can potentially jeopardize internal validity (13). Investigators do not need to supply the intervention in all sites in a short time frame. Those who wait provide control data during the time when others receive the intervention, reducing the risk of confounding by time-related variables. This often can result in stepped wedge designs taking longer to implement than other designs, and site differences and implementation processes can vary significantly over time. There is also the risk of contamination in later sites or intervention fatigue—both can wash out potential intervention effects (13). The study can be based on serial cross-sectional data collected by sites for different time periods (sites cross over) or by following a cohort of individuals over time (individuals cross over). This design can also be combined with other designs; we give an example in Table 3 where elements of this design are combined with a SMART design.

### Re-formulating Observational Studies as Pragmatic Designs

Answering research questions with pragmatic designs rather than relying on retrospective observational data requires a shift in thinking. Instead of asking retrospective questions about interventions that occurred in the past, the goal is to prospectively collect data about planned interventions in the future. As one example, Auger et al. (3) used interrupted time series analyses to assess whether US school closures were associated with decreased COVID-19 incidence and mortality. The study team gathered known timing of school closures and state-level data on COVID-19. The ultimate goal of the analysis was to determine whether schools being open or closed impacted the trajectory of the pandemic. In Table 3 (see example for “Stepped wedge design”) and Figure 5, we describe an approach using a pragmatic trial to answer a similar research question. Here, instead of retrospectively assessing what happened when schools closed, we delineate how we can instead assess what happens when they re-open using a combination of stepped wedge and SMART designs. The scope of this question is more local (depending on the scale of the pragmatic trial), although, as noted, pragmatic trials strive to be generalizable. Yet, it is also inherently more useful moving forward, as the decisions that need to be made now are fundamentally ones about re-opening. How to best re-open schools is not a question that can be answered by the previous observational analyses. Pragmatic trials are well-suited to answer questions that can more effectively guide future policy and generate information about how to best implement those policies in practice.

## Discussion

Robust evidence on what works to reduce transmission of SARS-CoV-2 is vital to protect people's health and welfare. However, it is clear there are key barriers to causal inference when studying interventions aiming to decrease morbidity and mortality from COVID-19 with observational, ecologic designs. Such designs are well-known to be susceptible to bias and we have explicated how this could lead to spurious conclusions. While we have suggested ways to strengthen internal validity to mitigate bias for these designs, we argue that re-formulating research questions to be answered instead by pragmatic trials would strengthen the evidence base to a greater degree. Further, pragmatic designs would also prioritize external validity and produce evidence to support implementation.

Studying large-scale interventions to prevent COVID-19 presents particular challenges. Thomson proclaimed that “the worldwide response to the COVID-19 pandemic may be the first truly global natural experiment of the modern, big data era” (34). Yet, the term “natural experiment” is somewhat of a misnomer. Policy responses being studied are not naturally occurring, but are decisions driven by the pandemic's trajectory and social and political will. As with all observational studies, the observational designs described above are at risk of confounding from unmeasured variables. A key issue we have highlighted is the difficulty of disentangling the effects of contemporaneous changes in policy to determine which was most effective. It is also challenging to correctly take into account the lag from the time a policy is put into place to when it is adhered to by a plurality of the public. Compounding this is the infectious nature of COVID-19 which necessitates building in additional lag time, varying according to outcome, to account for relevant incubation period, time to symptoms, or time to death. Finally, due to the dynamics of transmission, outcomes may be non-linear and require more complex modeling. Further, most studies have relied on pre-existing data, rather than prospectively collecting original data. This influences the types of outcomes and covariates that can be measured and analyzed.

It is possible to further improve the validity of observational studies by leveraging data at the individual level. This enables one to, at a minimum, avoid the ecologic fallacy. Instrumental variable analyses and regression discontinuity designs are robust methods to control for unobserved confounding, a key problem with observational data (35–37). Regression discontinuity designs, in particular, may be useful to study interventions for prevention of COVID-19 because identification relies on interventions being assigned based on thresholds. Many government policies have been allocated based on thresholds such as levels of COVID-19 incidence or geographic boundaries (22, 38). However, both regression discontinuity and instrumental variable analyses estimate the local average treatment effect, which has high internal validity compared to other estimands, but may not be the primary effect of interest (39). Furthermore, the local average treatment effect may only be generalizable to a subset of the population and may differ in magnitude from the treatment effect for the entire population (37, 40). It should be noted that regression discontinuity designs may also be applied prospectively, which has advantages including that outcomes can be measured before assignment (37) and data collection can be targeted to necessitate fewer observations (41). However, concerns about generalizability beyond the threshold values remain (40).

Given the limitations described, it would be useful to augment studies with other pragmatic trials outlined here, such as preference, SMART, and stepped wedge designs that have higher external validity. Such designs will require more planning and participant buy-in but could generate data that may be less susceptible to confounding than observational studies and be more “visible” as they occur, which may help improve uptake as well as promote acceptance of the findings. These prospective designs can be critical to identifying which interventions or components are most impactful, overcoming one of the primary challenges with the observational designs described. Importantly, these adaptable designs allow for population sub-groups that may experience disparities related to COVID-19 to be influential in the development and implementation of the studies. Preference designs, or example enable the determination of whether participants preferring one intervention have differential responses compared with participants preferring another intervention. This could aid municipalities and health systems in determining whether, in the face of resource constraints, it makes most sense to offer a one-size-fits-all intervention, to offer people choices, or to prioritize which delivery options work for certain groups. SMART designs could help clarify which interventions work in what sequences and combinations for what people and help throw out less useful components without waiting for the study to be fully complete. Stepped wedge designs offer a phased practical framework for a study to take place in real-world conditions with rapid scale-up logistics. They can be slowed down and sequenced in ways that capture useful information while scale up is occurring. As with all studies, is it important to consider threats to validity related to these proposed implementation-focused designs when planning them. The particular advantage of these flexible and responsive designs is that they specifically allow researchers and officials to study the implementation process for interventions and how to improve it moving forward.

As the evidence base grows, it is also important to understand if treatment effects vary across groups. Racial disparities in COVID-19 incidence, hospitalizations, and death are well-documented (42–44). In designing future studies, it is key to assess whether interventions are acceptable and effective for those at highest risk, in addition to the population as a whole. The pragmatic designs we describe can help to do this. Preference and SMART designs may enable recruitment and retention of populations that may be hesitant to join, thereby, increasing generalizability of results. Pragmatic designs in general can address feedback and be responsive to communities' concerns while determining what interventions work and how to best implement them in minority populations that suffer the highest burden of COVID-19. Being intentional about this can help address and narrow the gap (45).

In the face of a novel disease, people are unlikely to want to be test subjects for experimental interventions. However, the reality is that if we do not generate evidence before implementing a policy universally, we are all test subjects. The APEASE framework could aid in planning evaluations of non-pharmaceutical interventions to prevent COVID-19 that overcome people's reluctance to participate. Intervention components should be evaluated on: (1) acceptability to stakeholders, (2) practicability of implementation in its intended context and resources, (3) effectiveness and cost-effectiveness at achieving desired objective in the target population, (4) affordability at scale, (5) side effects or unintended consequences, and (6) equity between advantaged and disadvantaged sectors of society (46). These criteria offer a holistic assessment of the acceptability and feasibility of the intervention's implementation if evidence supports its utility.

## Conclusions

It is not possible or ethical to do large-scale randomized trials of all community interventions to reduce COVID-19. However, as we move forward in the arc of the pandemic, we must ensure that we are choosing designs that are of the highest validity possible. We have proposed use cases for pragmatic designs that could be implemented in the real world to strengthen the evidence base for critical decisions such as how to re-open schools safely. These designs can help us better understand what we should be doing, when, and for whom to prevent morbidity and mortality from COVID-19 and future epidemics.

## Author Contributions

JD and MH conceptualized the idea and did the literature search. JD, MH, KS, and CM wrote and revised the manuscript. All authors read and approved the final manuscript.

## Conflict of Interest

The authors declare that the research was conducted in the absence of any commercial or financial relationships that could be construed as a potential conflict of interest.

## Publisher's Note

All claims expressed in this article are solely those of the authors and do not necessarily represent those of their affiliated organizations, or those of the publisher, the editors and the reviewers. Any product that may be evaluated in this article, or claim that may be made by its manufacturer, is not guaranteed or endorsed by the publisher.

## Abbreviations

COVID-19: Coronavirus-19
ITS: interrupted time series
DID: difference-in-differences
SMART: Sequential Multiple Assignment Randomized Trial.
